# “The right time is just after birth”: acceptability of point-of-care birth testing in Eswatini: qualitative results from infant caregivers, health care workers, and policymakers

**DOI:** 10.1186/s12887-020-02242-2

**Published:** 2020-07-15

**Authors:** Emma Sacks, Philisiwe Khumalo, Bhekisisa Tsabedze, William Montgomery, Nobuhle Mthethwa, Bonisile Nhlabatsi, Thembie Masuku, Jennifer Cohn, Caspian Chouraya

**Affiliations:** 1 Department of Global Health, George Washington University School of Public Health, 950 New Hampshire Ave, NW, Washington, DC, 20052 USA; 2 Elizabeth Glaser Pediatric AIDS Foundation, Mbabane, Eswatini; 3 Ministry of Health, Mbabane, Eswatini; 4 Elizabeth Glaser Pediatric AIDS Foundation, Geneva, Switzerland

**Keywords:** Birth testing, HIV/AIDS, Point of care, Postnatal care, Qualitative, Interviews, Eswatini

## Abstract

**Background:**

Testing for HIV at birth has the potential to identify infants infected in utero, and allows for the possibility of beginning treatment immediately after birth; point of care (POC) testing allows rapid return of results and faster initiation on treatment for positive infants. Eswatini piloted birth testing in three public maternities for over 2 years.

**Methods:**

In order to assess the acceptability of POC birth testing in the pilot sites in Eswatini, interviews were held with caregivers of HIV-exposed infants who were offered birth testing (*N* = 28), health care workers (*N* = 14), and policymakers (*N* = 10). Participants were purposively sampled. Interviews were held in English or SiSwati, and transcribed in English. Transcripts were coded by line, and content analysis and constant comparison were used to identify key themes for each respondent type.

**Results:**

Responses were categorized into: knowledge, experience, opinions, barriers and challenges, facilitators, and suggestions to improve POC birth testing. Preliminary findings reveal that point of care birth testing has been very well received but challenges were raised. Most caregivers appreciated testing the newborns at birth and getting results quickly, since it reduced anxiety of waiting for several weeks. However, having a favorable experience with testing was linked to having supportive and informed family members and receiving a negative result. Caregivers did not fully understand the need for blood draws as opposed to tests with saliva, and expressed the fears of seeing their newborns in pain. They were specifically grateful for supportive nursing staff who respected their confidentiality. Health care workers expressed strong support for the program but commented on the high demand for testing, increased workload, difficulty with errors in the testing machine itself, and struggles to implement the program without sufficient staffing, especially on evenings and weekends when phlebotomists were not available. Policymakers noted that there have been challenges within the program of losing mothers to follow up after they leave hospital, and recommended stronger linkages to community groups.

**Conclusions:**

There is strong support for scale-up of POC birth testing, but countries should consider ways to optimize staffing and manage demand.

## Background

Despite advances in prevention of mother-to-child transmission of HIV, many infants are still born infected with the virus. Untreated pediatric HIV has a peak mortality at 2–3 months of age, and half of HIV-infected infants will not survive past age two without treatment [1]. Although there are limited options for HIV treatment for infected neonates, new medications are in the pipeline and will be available soon. Thus, understanding the acceptability and feasibility of HIV testing at birth is an important first step prior to the introduction or scale up of birth testing programs, which may occur in more countries in the near future.

The World Health Organization (WHO) recommends considering the addition of birth testing to national programs where the 6-week testing program is strong [2]. Testing at 6 weeks of age was deemed to be optimal from a public health perspective, in order to capture the greatest number of infants who were infected in utero, during delivery, and during breastfeeding. The 6-week mark also corresponds well with most country’s immunization schedules, and caregivers often access health care around that time so that infants receive childhood vaccines [3]. However, for some infants - many infected in utero - waiting until 6 weeks is too late as they will have already developed advanced disease or not survived. The consequences of untreated HIV are especially severe among preterm and low birth weight infants [4]. Testing immediately after birth is essential for identifying infants infected in utero and starting them on treatment as soon as possible.

Very few countries have introduced testing at birth. Targeted testing based on maternal risk factors has been piloted in a number of countries in southern Africa, including Zimbabwe and Malawi [5–7]. South Africa introduced deoxyribonucleic acid (DNA) polymerase chain reaction (PCR) birth testing for all HIV-exposed infants in 2015; preliminary data showed high acceptance of testing at birth, but challenges in coverage of subsequent testing for those who tested negative, and issues with linkage to care and medication adherence for those testing positive [8–10].

Eswatini was an ideal location for birth testing because of the high proportion of infants who are delivered in health facilities (94.0%) [11], the strong 6 week testing program (87.7% of HIV-exposed infants tested) [12], the high prevalence of exposed infants (close to 10,000 HIV-exposed infants are both annually) [11], and the availability of point-of-care platforms. With funding from Unitaid, the Elizabeth Glaser Pediatric AIDS Foundation (EGPAF) worked with the Ministry of Health of the Kingdom of Eswatini to pilot POC birth testing in three health facility maternity units, starting in 2016, building on an existing program of early infant testing using POC platforms. POC testing has been shown to reduce median turnaround time of results at 6 week testing from 55 to 0 days [13] and has also been shown to be feasible for testing at birth [14, 15]. The opportunity to offer POC birth testing allows most caregivers to receive results before they are discharged from the hospital after delivery, or shortly thereafter, and thus can improve the survival chances of those found to be infected by starting treatment earlier. The introduction of POC birth testing in Eswatini provided a unique opportunity to conduct both quantitative research on testing uptake and qualitative research on acceptability among various stakeholders.

Of the three POC platforms used for birth testing in Eswatini, two of the platforms were exclusively used for birth testing; one platform was also used for standard 6-week testing; all were placed in laboratory rooms inside of hospitals with delivery wards. Tests were typically run by phlebotomists during the week, and nurses at nights and on weekends. All HIV-exposed infants born in the pilot facilities or presenting to the pilot facilities within 3 days were eligible and offered a birth test. This study was aimed at understanding the acceptability and feasibility of POC birth testing among caregivers of HIV-exposed infants, health care workers, and policymakers in Eswatini. It was part of a larger study of POC birth testing which was conducted in the three highest volume maternity settings in the country.

This is one of the first studies in the world to offer POC birth testing in a routine setting, and by offering birth testing at these high-volume sites, this program covered over 30% of births in the country [16]. In addition to clinical data being collected to assess effectiveness, which is published separately, it is important to understand the acceptability, feasibility, and contextual barriers and facilitators in order to smoothly introduce and sustain POC birth testing. The goal of this study was to understand the experience of caregivers, health care workers, and policymakers with respect to point-of-care birth testing in Eswatini. The study aimed to assess the feasibility and acceptability of this type of testing, as well as identify possible barriers, concerns, or program support needs that should be addressed or implemented along with birth testing.

## Methods

### Data collection

This study was the qualitative component of a larger study on birth testing in Eswatini, which also collected quantitative data. The number and type of respondents was purposively selected to represent a range of different stakeholders, namely caregivers, health care workers, and policymakers, and enough respondents to capture potentially divergent views. Health care workers at each of the three health centers offering birth testing were invited to participate, as were policy makers at the national and regional level involved in pediatric HIV programming. Caregivers whose infants were offered birth testing were invited to participate, regardless of whether they accepted testing or not.

Interview guides were developed for this study and pre-tested before use (Supplemental File 1). Interviews were conducted in either English or SiSwati, depending on the preference of the respondent. Interviews generally lasted between 30 min and 1 h, and were held in private locations. When possible, interviews with caregivers were scheduled for days when they had clinical appointments to reduce travel burden. Participants were compensated for travel costs. Interviewers used field guides to ensure that all relevant topics were covered, but allowed respondents to focus on the issues most important to them. Transcripts were translated to English if necessary, and transcribed verbatim, by bilingual research assistants. Study team members reviewed a selection of transcripts to check for quality.

### Data analysis

Data were analysed in MaxQDA (Version 12). Transcripts were read for familiarity and coded using a priori codes developed based on the study objectives. Very minor adjustments were made to the code list during the coding process to fully capture the responses. Content analysis and constant comparison were used to identify key themes arising from each type of respondent [17]. Responses were organized by theme and sub-theme, and illustrative quotes identified for each.

### Ethical approvals

This study was approved by the Advarra (formerly Chesapeake) IRB in the US, and the Eswatini Health and Human Research Review Board (EHHRRB). Participation was entirely voluntary, and participants gave written informed consent before participating in interviews.

## Results

A total of 52 interviews were completed: 28 caregivers of infants who were offered POC birth testing, 14 health workers involved in pediatric HIV testing, and 10 policymakers. There were no refusals to participate, among those invited.

Results are organized into six themes: knowledge about POC birth testing, experience with POC birth testing, opinions about POC birth testing, barriers and challenges to the use of POC birth testing, facilitators to the use of POC birth testing, and respondent suggestions for improving a POC birth testing program. Results are presented by theme, including responses from caregivers, health workers, and policymakers.

### Knowledge about POC birth testing

Overall, respondents were accepting of birth testing and understood its benefits. Caregivers had a basic understanding of infant HIV testing, but both caregivers and health workers spoke specifically about how infant HIV testing can help children live longer. Caregivers felt that there were important advantages to HIV testing at birth, and desired testing at local facilities, in order to make it more accessible to more people. Caregivers were aware that testing could be conducted quickly, and understood the benefit of starting treatment quickly.

*“I think the right time is just after birth, just like I gave birth yesterday and he got tested after birth … it will help me in taking care of him … because if I now know that for instance he got the virus, I have to take care of him.” (Mother, #02)*

Caregivers stated that they got a lot of their information about HIV birth testing from the nurses in antenatal care, and for the most part, felt that they received accurate and timely information. Caregivers were very influenced by the encouragement of HIV counselors, and some likened a negative HIV test result to a “reward” for adhering to their HIV medications or other recommendations from the counselors.

*“... I will say, this is a good thing to do. If you follow well what they told you to do in the [antenatal] clinic, you are able to see … that the child can come out clean [without HIV] … ” (Caregiver, #17)*

Caregivers who had a positive experience felt that they would be able to share their experiences with others and could provide information and recommendations to other families.

Policymakers were very knowledgeable and enthusiastic about POC birth testing, commenting on the benefits of being able to return results to caregivers prior to discharge, and the ability to start infants on ART regiments quickly, to reduce mortality risk.

*“those children who are positive … we are worried that they may die if they do not get treatment … the use of Point of Care to implement birth testing … reduces the time for sample transportation to the Lab and sample results return to the facility and been provided to the caregiver meaning that actually the mother should be able to go home knowing whether the child is HIV positive or negative … ” (Policy maker, #03)*

*“So in terms of what is possible now which was not possible before … we are able to test and start this children on treatment on exactly the same day. From a public health perspective in the long term; we going to reduce infant mortality.” (Policy maker, #05)*

### Experience with POC birth testing

For most caregivers, their perception of their birth testing experience depended on the test result. Most of the caregivers who received a negative infant HIV test appreciated the rapid return; those who received a positive infant HIV test did not express a specific desire to learn the positive status more quickly. However, caregivers did understand the value of the immediate HIV test and preferred to get results (and thus be able to begin treatment) rather than have a sick child.

*“It helps because as the child’s parent as your spirit is free knowing that your child is healthy. Even when the child is not, you get help and move on...” (Caregiver, #13)*

*“I won’t say I’m happy because the results are not yet back. So I don’t know what they will say … so if they will come back positive … I won’t keep quiet [will be upset] … ” (Caregiver, #12)*

Caregivers stated that they had reduced anxiety when they received results faster, as waiting for the test result was one of the difficult aspects of testing, and caused a lot of stress.

Caregivers had very mixed experiences related to family support. While most women delivered without a partner, and many without another family member, some reported having strong support systems at home. Caregivers had less anxiety and fear about birth testing if their families knew their status and were supportive, including encouraging them to adhere to medications. Those who did not have supportive or informed family members felt that a positive infant HIV birth test would have significant negative implications for them and their child.

*“She [my mother] would encourage me to go to the hospital, take my treatment...and encourage me that life will continue.” (Caregiver, #03)*

*“It is very difficult such that even if he [the child] was positive, there is no one you can tell... even me there’s no one knowing at home that [I] am in such a situation...so it is difficult that.. I don’t see that they will ever know...it will be just my issue.” (Caregiver, #02)*

Most of the caregivers seemed to understand the messaging around HIV birth testing, and credited the HIV counselors at the facilities for providing accurate and non-judgmental information. The caregivers expressed appreciation for health facility staff who were helpful and informed and they specifically commented on the staff members who took extra care to be discreet and not reveal their status or the status of their infant to others.

*“No I was not scared of being discriminated because the nurse didn’t say ‘hey you … let’s go and test the child’ … she called me, she called me nicely as if there is something she wants to ask on the side....even if there was a relative inside I wouldn’t be … because the nurse didn’t say ‘let’s go and check the child’s status’...now, there was no discrimination.”(Caregiver, #13)*

*“Yes, they were able to counsel me* … *Nurses were very good, they were friendly, they did not shout at me, they answered all my questions I had and I was not afraid of anything* … *I am happy about them.” (Caregiver, #04)*

Some caregivers held the opinion that newborns seemed too small or fragile for testing because the heel-prick would be uncomfortable or painful. However, even those who said they were scared during the blood draw understood the importance of testing (even if they did not understand why a heel-prick was necessary, as opposed to a saliva sample). There were a few caregivers who had negative experiences with health facility staff, and felt they were not treated well, which worsened their overall experience of testing.

Health care workers mostly reported positive experiences with POC birth testing, with the exception of the increased workload, and the exacerbation during shifts with staff shortages. Phlebotomists reported working extra hours to keep up with demand, especially where the POC platform was used both for birth testing and routine infant testing (e.g. 6 week infant virologic testing). Nurses appreciated receiving training themselves, as well as when more members of the staff were also trained, in order to provide better coverage during periods with fewer staff members working. Originally phlebotomists ran all of the tests. However, nurses specifically requested training on the platform and a change to the program was allowed, to certify nurses to run the POC machine, and this change was applauded by nurses especially for the benefits of being able to run the tests on weekends.

*“We were taught well and we understood, we understood and we saw its importance. The problem [before] was staffing … ” (Health care worker, #04)*

Health care workers also appreciated when mothers had received counseling in antenatal care, and generally felt that the coordination across antenatal and postnatal care was smooth, and contributed to a positive experience with testing.

Policymakers were very positive and enthusiastic about the POC birth testing program. However, they had mixed experiences in training staff at health care facilities: some stated that trainings were productive and successful, while others thought the trainings were challenging, especially in the context of limited resources.

*“The health workers themselves must be convinced about it and there is no way other than providing information or re-orientation of the health workers - especially the nurses - on benefits of birth testing. Once health workers believe in that, they can take full control and also convince their clientele.” (Policymaker, #02)*

Policymakers stated that birth testing presents a challenge for mothers in that there is fear in knowing the HIV status of the infant. However, they did not see this as different from testing at other ages nor as a reason to not offer testing, but rather a challenge to be addressed as part of the birth testing program.

### Opinions about POC birth testing

Almost all of the caregivers expressed favorable or positive opinions about birth testing, although those whose infant received a positive HIV test result expressed more mixed opinions.

Caregivers not only found the reduction in timing to be personally beneficial in reducing anxiety, but some said that they would recommend birth testing to others in the community. Those who felt that they had a positive experience wanted to share the importance of testing and encourage other mothers.

*“I can tell other woman that it is very true that you can give birth to a child without HIV yet you have it … … if you listened to information from the health care workers [about prevention and testing] … ” (Caregiver, #10)*

Mothers who received negative results had a positive experience because, in addition to the health benefits for the infant, they also saw the negative result as a “reward” for adhering to the counselors’ and nurses’ recommendations and felt a sense of pride.

Health care workers were in favor of POC birth testing because they believed it was good for the health system, even though it created more work for them individually. Many stated that birth testing was needed, given that many positive infants were not identified until they were older and had more advanced disease. Health care workers were especially complimentary about POC platforms, with many stating that they were sorely needed by the health system in order to fill the high demand for testing.

*“We benefit from knowing if the child is born with HIV/AIDS … So now that we are able to do birth testing, we can early identify the children that are HIV positive so they can get access to early care and treatment/ART. So they can boost their immune system and live a long life and reach pass the six weeks most children didn’t.” (Health care worker, #06)*

Health care workers expressed support for national scale up of a POC birth testing program and they agreed that all health facilities would be able to handle POC birth testing, but that more staff would be required at many facilities.

*“Definitely … it is more of an advantage than a disadvantage … it is really a comfort for a mother to go home knowing status for the child … it is really helping in the scourge for HIV … [and] with the management of HIV in our country … ” (Health care worker, #03)*

Health care workers inferred that caregivers appreciated POC birth testing and were largely in favor of the program. Only one health care worker reported a case where mother was very opposed to testing, but most found their patients to be very accepting of testing.

Overall, policymakers expressed favorable or positive opinions about POC birth testing. A few policymakers specifically expressed a desire to scale-up birth testing across the country, but noted this would require more political and economic investment. Policymakers also wanted to ensure that the addition of birth testing to the algorithm did not interfere with the 6-week testing program, but were encouraged that health workers were stressing the importance of subsequent testing to mothers whose infants received a negative birth test.

### Barriers and challenges to the use of POC birth testing

For caregivers, the largest barrier to the use of birth testing was negative family influence. If family members were unaware of their status or opposed to testing, they were less comfortable with accepting birth testing. If they were accompanied by a family member, that particular person’s knowledge of their status or opinion about testing could be a large influence, and a potential barrier.

Health care workers identified many challenges to the POC birth testing program, the primary one being the limited number of staff available to run tests. Nurses felt the extra burden, adding to an already heavy workload (especially on weekends), and some expressed their preference to have one person dedicated to birth testing alone, rather than all of the nurses trying to navigate it along with other duties. They also suggested having more POC platforms per facility, in order to meet the high demand of testing.

*“The ward capacity is usually full and we even have floor beds during those times so the birth testing is taking a back seat … because it is not what we are here for … primarily we here to help mothers give birth … to monitor the progress of labour … and we under staffed a lot … ” (Health care worker, #03)*

*“There are [a] lot of women that need to do birth testing here in [redacted name] Hospital. You see? It’s a lot. You have to do your nursing duties besides the birth testing and also do the birth testing. So you find that a lot of the children are missed.” (Health care worker, #06)*

Although the test itself was free to patients, and convenient for those already in the facility for delivery, high demand led to long queues and a backlog of tests of 1 or 2 days, especially over the hours with fewer staff members to run tests. Health care workers expressed concerns that some caregivers were asked to delay discharge in order to receive test results, which they did not feel was ideal. This was particularly challenging in the case of one non-government owned facility which charged a fee for an extra “inpatient” day if discharge was delayed. This cost, or the fear of this cost, was a challenge noted by a few health care workers.

A few health care workers said they felt that the POC test still takes too long, and they are not able to accommodate all of the mothers when the facility is busy. Some noted the frustration of having machine errors and having to run a sample multiple times, further delaying the receipt of results. Many health care workers worried about losing patients to follow up if results were not available until the next day, after the patient had been discharged, noting that many of the patients did not have working phones. One health care worker also noted that the guidance for testing of preterm infants, or those transferred from other facilities after 72 h was not clear.

*“You see some mothers end up leaving their results as fathers are there to fetch them … you see they leave the results.” (Health care worker, #01)*

*“Sometimes you are doing this test and it is not the only woman that you will be running the test for … Some are waiting for you, they are discharged, their relatives are waiting for them outside and they do not understand what is happening, why you are delaying her. Because you tell the person that it will take an hour. After an hour that person is expecting you to come back with the results, at time you do not come with the results you come back with sad news that ‘oh no my sister your results did not come out … let us start again’ you see.”(Health care worker, #03)*

Health care workers felt that the trainings were helpful, but that they simulated ideal operating situations. In reality, health care workers are challenged by more chaotic environments and sizable patient demands. They noted the challenge of health care worker turnover at a given facility, and the lack of trainings for new staff members.

*“The problem was staffing … who will do this thing? … we have a lot of services … [and] we are few.” (Health care worker, #05)*

Policymakers noted the challenges related to the extra burden on health facility staff, but they did not see this as much of a challenge as the health care workers themselves. They noted that there are staff shortages across the Ministry of Health, not only related to HIV services, and felt that facilities should be able to be more productive with their current staffing.

Some policymakers felt that by referring to the birth testing program as a “pilot,” it created a challenge among patients, who would not know if they could expect this service. Many felt that referring to the program as the “standard of care” would improve uptake among caregivers. Further, because the birth testing program relies on women giving birth at health facilities, a few policymakers noted the ongoing challenge to increase coverage of institutional delivery to 100%.

### Facilitators to the use of POC birth testing

Caregivers benefited from and appreciated supportive counseling, especially during antenatal care. Caregivers mostly felt that the counseling was clear and understandable, and they complimented those nurses who were discreet and protected their privacy. Caregivers also indicated wanting to “please” the nurses by adhering to recommendations. Thus, a good relationship with the nurses facilitated acceptance and uptake of birth testing.

*“If you are a mother who has the virus you get help the same way like everyone else … even a mother who does not have the virus yet ...she is also taught about the virus … because it happens that you end up giving birth without having the virus but when you check ...you find that you have the virus so when you go to the clinic you have knowledge it becomes easy to accept your situation that has changed...because you know, you have been taught well about the thing...about the virus.” (Caregiver, #13)*

Birth testing was also facilitated if the family was supportive and aware of the mother’s status. In cases where the family already encouraged the pregnant woman to take medications, testing of the infant would be seen as a “natural” extension.

*“I can say, the child’s father is supportive … he said I should agree to do everything they say [at the clinic]. When they check him [the child] we will accept every situation we face…because the child is ours so you can’t say, as I was able to accept, he is also my child I will be able to accept…He is very supportive.” (Caregiver, #16)*

Caregivers felt that the accessibility of POC enabled the uptake of birth testing because the test was at the same location as the mother. Since the mothers did not have to travel and incur additional costs, they saw the benefit of a convenient test.

Health care workers felt enabled to encourage and run POC birth tests when the facility was not “chaotic.” During shifts with sufficient staff levels (generally during the daytime, during the week), health care workers were better able to carry out tests. Birth testing was also facilitated when all of the health care workers in a facility felt competent to run the tests. Preliminarily, only phlebotomists received training, but these skills did not transfer to the nurses. Once these nurses received direct POC training, they were able to carry out more tests (especially on weekends and evenings), which facilitated further uptake of testing.

### Respondent suggestions for improving the POC birth testing program

Caregivers did not have specific suggestions to improve the POC birth testing program, except to make it available at more local clinics. Health care workers had a number of suggestions, most requiring more resources: they desired more platforms per facility, in order to get results back to facilities faster even when there was a backlog; they wanted more staff to be able to run tests; and they wanted more continuity between the weekday and weekend staff.

“*The problem … is the shortage of staff … you find that if the Phlebotomist is off during the weekend then the nurse who is remaining alone for the morning shift … she cannot test all the mothers … but if the staff was good enough, … the other one can continue doing the routine activities for the ward and the other one can concentrate on the Birth Testing.” (Health care worker, #03)*

*“Yesterday which is Friday, they did 7 caesareans … I have to go and see the caesareans and do rounds, also here is the birth testing - so we have the challenge … during the mid-week it’s better…the difficulties are during weekends.” (Health care worker, #04)*

*“We do have challenges eh … as nurses we have a challenge of being short staffed…in this program of birth testing, it is a lot of work I do not want to lie.” (Health care worker, #06)*

Policymakers felt that staffing shortages were a consistent challenge, and facilities needed to improve their efficiencies and workflows to improve services given the resource and staffing constraints which were unlikely to change.

*“So other facilities ,what they need to do is to have a paradigm shift and their attitudes because whenever you try to introduce a new service line the first thing they say is we under staffed … and then staffing will always be a challenge, so my view is … all other facilities need to see how best they can implement such an valuable service line with the capacity that they have at hand and integrated into their HIV testing clinic flow within the facility to see what works and to see how best to modify the clinic flow to be accommodated.” (Policy maker, #06)*

Health workers stated that more education is needed for families when a child is identified as positive, in order to link them to treatment, and educate them on the importance of initiation and adherence.

Policymakers gave many suggestions, both large and small scale, for improving the POC birth testing program. Policymakers felt that it was important to introduce POC birth testing as a standard of care, rather than a pilot program, so that it was understood to be routine. There were numerous recommendations to increase the involvement of community health workers, in order to maximize their influence in the communities where they directly work.

“*The Community-Rural Health Motivators, they should be vigilant, if they know that there is a child who was born at home and still not going to the clinic they should encourage the mother to go to the clinic to get services which is the BCG [vaccine] and the birth testing as well.” (Policymaker, #10)*

Policymakers suggested a need for new strategies to increase communication and education to men and male partners, to inform them of the benefits of POC and infant HIV testing, and increase their responsibility in the birth testing process in general.

*“I think information and education is key in this regard and the involvement of men in the care of their children so that they know the benefits, and they weigh their benefits versus not testing… the responsibility of bringing the child and ensuring the child is safe lies on both parents the women and men but however the women are usually in control even though they do not have the authority to make the decisions.” (Policymaker #02)*

Many respondents recognized that when a new service is introduced, health facility staff may feel that it is an added burden, especially if they are already short staffed; a few respondents suggested instead that facilities need a “paradigm shift” toward focusing on improving skills of current staff and involving the “whole team” from front desk to clinicians. Most respondents agreed that it was crucial to secure “buy-in” from the entire team or facility when introducing POC birth testing.

While most respondents had suggestions to improve the program, most focused on being able to serve more people even more quickly, and almost all felt that the POC birth testing program should be scaled up to the entire country.

## Discussion

Overall, most respondents had positive experiences with point of care birth testing. Caregivers appreciated receiving birth test results quickly, and health care workers saw a huge benefit in delivering results to mothers before they were discharged, rather than needing to return to the facility. It was difficult for caregivers to disentangle feelings about the test results themselves from the actual experience with testing, and the main influence on their experiences and willingness to test was having supportive and informed family and social support. While POC testing reduced many of the cost and distance issues, respondents expressed continuing concerns around travel time, especially for those who still live far from POC testing facilities. Even with POC, health care workers noted continued issues with wait times, especially in facilities with high demand, long queues, or frequent errors requiring retesting. Health care workers stressed the increased workload, and especially noted the challenges during shifts with staffing shortages, such as nights and weekends. However, when considering the benefits, health care workers and policymakers were supportive not only of existing POC birth testing, but national scale up.

Similar to the few other countries with qualitative assessments of birth testing programs, this study found the program to be largely acceptable, but with concerns about the additional infrastructure burden, as well as potential loss to follow up for future testing or linkage to care for infants testing either negative or positive. There is very limited country experience with birth testing; however, in South Africa, the demands on the health system were described as requiring large financial investments, “meticulous, planning, innovation and supervision,” as well as a plethora of new tools, standard operating procedures, trainings, monitoring, and support [18]. Pilots in Thailand, Kenya, Zimbabwe, and other countries may shed additional light on various implementation challenges and solutions.

This study confirms the views of participants found in other studies on POC, for example from Kenya and Zimbabwe, where the reduction in waiting time for HIV test results reduced parental anxiety [19, 20]. Some participants expressed fears about infant pain from the heel-prick and newborn fragility, but it did not seem to deter them from testing. Other studies have shown increased parental satisfaction with pain management when they were able to comfort the infant, so the ability to draw the sample with the caregiver holding the infant likely alleviated some anxiety [21]. While caregivers had good understanding about most aspects of infant HIV testing, they did not understand the reasons for drawing blood from the heel; this lack of understanding may have contributed to their feelings that the heel-pricks were excessive or too painful.

This study confirms other studies showing that POC is acceptable and even preferable for many health workers, but they have concerns about capacity, additional workload, and sustainability [22]. Many health workers felt that demand outstripped the ability to provide rapid results, especially on weekends and nights when there were fewer health workers available. Many health workers stated that additional platforms or use of platforms with multiple modules in a facility could help, in order to run multiple tests at once. However, other strategies, such as hiring phlebotomists to work overnight or on weekends, or instituting triage systems, may also be useful and potentially more practical and cost-effective.

More education and support are still needed, especially on the importance of treatment for positive infants, and about linkage to and continuation in care. Acceptance of birth testing was high, but quantitative data indicate a lower proportion of caregivers whose infants are immediately (within 14 days or diagnosis) started on ARVs [14]. This could be for multiple reasons, including the ease of an onsite test with no travel; the fact that the test is a one-time event unlike medications; the ability to test in secret without other family members; and that many women delivered alone and may have had autonomy to accept testing where they may not be able to accept medications once other family members are involved.

While mothers who received negative infant HIV test results reported positive experiences with testing, mothers who received positive infant HIV test results often had negative experiences, which may deter future care seeking or medication adherence. Care must be taken to ensure that health workers do not blame mothers or families for unfavorable test outcomes and families are treated respectfully and encouraged to stay engaged in health care. The importance of family support in acceptance of testing and medication adherence may also be important in early identifying women and infants at high risk for being lost to follow up for testing and treatment.

### Strengths and limitations

As this study did not have a comparator arm, respondents did not generally have multiple experiences with testing to compare, either between birth testing POC vs. conventional, or POC birth testing vs. POC 6 week testing. However, some respondents had previous children who had received a conventional test at 6 weeks or older, or other experiences with HIV testing. Health workers and policymakers generally had previous experience with or knowledge of conventional testing, and were able to comment on differences with POC testing.

Eswatini is unique in some cultural traditions (for example, higher prevalence than other countries of mothers wanting to gain consent of husband before accepting HIV testing or treatment of infants) and health system arrangements (for example, the reliance on phlebotomists); thus, while there are many similarities to other under-resourced health settings, these findings may not be entirely generalizable to other settings.

This study provides important information about the acceptability of POC birth testing by including the views of a range of stakeholders: caregivers, health care providers, and policymakers. This was a first study to explore the views of primary stakeholders; future projects may wish to expand to include views of other stakeholders, such as additional family members or community health workers, who may also be involved in birth testing.

This study complements previous quantitative findings from the same overall study [14], and is a critical first step in understanding the value and feasibility of birth testing.

## Conclusions

Point of care birth testing is largely acceptable to patients, providers, and policymakers, but some issues still persist around testing anxiety and lack of social support from the patient side and about necessary increased capacity and workload for POC from the system side. Families mostly had positive experiences with birth testing, and with few exceptions, appreciated and were grateful for helpful and non-judgmental staff, especially nurses, who should be recognized for the important work they are doing to increase testing. However, both patients and providers were concerned about follow up after testing, and efforts need to be made to ensure that, especially those who test positive, are linked with care and supported through treatment options. In countries with strong 6 week testing, large proportions of facility births, and a sufficient health workforce, POC birth testing could be an important addition to save infant lives.

## Supplementary information

**Additional file 1.**

## Data Availability

Tools are available upon request from the authors. Data contain potentially identifiable information and cannot be shared outside of the study team; however summarized reports can be shared on request.

## Abbreviations

AIDS: Acquired Immunodeficiency Syndrome
ARV: Antiretrovirals;
EID: Early Infant Diagnosis
HIV: Human Immunodeficiency Virus
POC: Point of Care
WHO: World Health Organization
