# Supplementary material for: “The right time is just after birth”: acceptability of point-of-care birth testing in Eswatini: qualitative results from infant caregivers, health care workers, and policymakers
Source: BMC Pediatr. 2020 Jul 15;20:347. doi: 10.1186/s12887-020-02242-2 (PMC7362515; doi:10.1186/s12887-020-02242-2)
Supplement: Supplementary file 1 — Additional file 1. [file 12887_2020_2242_MOESM1_ESM.zip › Feasibility of POC BT IDI Guide_Mothers_Ver 2.0 EnglishR2.docx]

**IN-DEPTH INTERVIEW GUIDE FOR MOTHERS/CAREGIVERS**

Date of Interview: ­___ ___ / ___ ___ / ___ ___ Interviewer ID:

*Day/Month/Year (e.g. 22/01/18)*

Facility Code:

Participant has signed written consent for this interview? Yes No

Participant agreed to be audio-recorded? Yes No

*“I would like to ask you about your experiences and opinions on testing of infants for HIV at birth. Our discussion will also include your use of such services and experiences you may have encountered at home after having the baby tested for HIV.* *You can talk freely about your experience and opinions while I take notes. Please remember that you do not have to answer question you do not wish to answer. May we begin?”*

For all mothers/caregivers

1. Please tell me what you know about testing children at birth?

***Probe:*** When is the right time, after delivery, to test children for HIV? Why is it important for children to be tested for HIV soon after delivery? What is your personal opinion about testing children for HIV soon after delivering? Where can such services be received within Eswatini?

For mothers/caregivers who **TESTED** their children for HIV at birth or within 3 days after delivery

1. You said you had your child tested for HIV at birth or within 3 days after delivery, please tell me about your experience with testing your child for HIV at this time?

**Probes:**

Allow the mother/caregiver to tell their story. Thereafter probe using the following questions where appropriate and/or if not mentioned:

- How did you feel about testing your child for HIV at this age? *(Probe for reasons behind the feelings; what made you feel this way)*
- What made you decide to test the child for HIV at this age?
- What were your thoughts, feelings about the services provided (including counselling and specimen collection): were they good or bad; were you happy or not happy about them and give reasons?
- How easy was it for you to decide to test the child for HIV at this age? *(Probe for the role of fear of stigma, fear of partner violence, HIV positive status disclosure, age of the child, quality of services at health facilities, etc.?*
- What were the nurses/HCWs like? *(Probe on their interaction with the nurses, that is, if the HCWS were friendly, approachable, helpful, supportive, etc. If they addressed her questions/concerns, provided adequate counselling, etc.)?*
- Did you have to stay longer than expected at the facility? Why do you think so? Did this cause additional challenges?
- When you got back home/your family what kind of support you and your child received from your husband/partner, the father of the child and other family members?
- How did you disclose that the child has been tested for HIV and the child’s HIV status?
- What was the reaction of the father of the child upon learning the HIV status of the baby?
- What was the reaction of other family members upon learning the HIV status of the baby?
- If you did not receive any support would you tell us what happened and how it was addressed?
- Did anything change in the way you take care of your child after testing them at birth? *(Probe on issues such as breastfeeding)*
- Is there information that you would have liked to be given when your child tested at birth that you think was not provided? (*Probe on how, where and by whom they would like to receive such information*)
- For **those whose children tested HIV+ at birth**:
- Did you have issues accepting the results? If yes, how long did it take to finally accept the results?
- Has your child been initiated on ART? If yes, what barriers did you have to overcome to initiate the child on ART? What support did you receive from your husband/partner, father of the child and other family members about initiating the child on ART.
- Did you have any concerns about giving the medicine? *(Probe for issues around taste, dosage, secrecy)*
- If you have already started treating the child, have you encountered any challenges in giving the medication? *(Probe for issues around taste, dosage, secrecy)*
- For **those whose children tested HIV- at birth:**
- *If it has been more than 6 weeks since the birth*; did you have the child tested again at 6-8 weeks?
- *It re-tested at 6-8 weeks;* compared to testing the baby at birth how long has it taken you to receive your results for the 6-8 weeks testing?
- What can you say about the two tests; testing at birth vs testing at 6 weeks? What are the benefits to the two test?
- How are you planning to take care of your baby that now you know the 6 weeks HIV results?
- If not retested the child at 6-8 weeks; what made you decide not to test the child again?
- What are your plans to have the child tested in future?
- *If it has been less than 6 weeks since the birth*, do you plan to have the child tested again at 6-8 weeks? If not, why not?
- *All women whose children tested HIV- at birth;*
- What measures have you taken to keep your child HIV free?
- Do you feel you were given enough information about breastfeeding?
- Do you feel you were given enough information about your medication?
- What do you think has been the benefits of having your child tested for HIV at birth or within 3 days after delivery?
- Do you feel that you did well by having your child tested for HIV at birth or within 3 days after delivery? Why/why not?
- If you were to go through this process again would you decide to have your child tested for HIV at birth or within 3 days after delivery? Why/why not?

***Go to Question 5***

For mothers/caregivers who **DID NOT** test their children for HIV at birth or within 3 days after delivery

1. You said your child was not tested for HIV at birth or within 3 days after delivery, please tell me why you did not decide to test your baby for HIV at this time?

**Probes:**

First allow the respondent to list the reasons. Note each reason and probe for more information on each reason. If not spontaneously given ensure to probe on need for the father’s consent, fear of a HIV positive result, HIV status disclosure, alternative treatment, etc.

1. What could the clinic have done to make it easier for you to decide to test your child for HIV at birth or within 3 days after delivery?

**Probes:**

First allow the respondent to list what the health facility could have done. Note each suggested action and probe for more information on each.

1. Are you planning to have the child tested later?

**Probes:**

First allow the respondent to explain their thinking. Then ask about when they plan

to get the next test, why they prefer the later test, what information they would

have wanted about the different test times.

1. If the baby **was tested at 6-8 weeks**, what motivated you to test the child for HIV at this point? How did you perceive the 6-8 weeks’ test to be different (good or bad) from the test at birth?
2. If the baby **was not tested at 6-8 weeks**, what made you decide not to test the child at this point. **Probes:** When are you planning to test the child? Considering that the child is at risk of being infected with HIV. What are your plans in the future for taking care of the baby to ensure that they do not acquire HIV?

For all mothers/caregivers

1. Do you have any suggestions on how testing of children at birth or within 3 days after delivery can be improved?
2. Based on your experience with testing children for HIV at birth or within 3 days after delivery, would you recommend birth testing to other mothers/caregivers? Why/why not?
3. Based on your experience with testing children for HIV at birth or within 3 days after delivery, would you recommend that the country provides these services in more health facilities? Why/why not?
4. Is there anything else you would like to share with us about your experience with birth testing?

*Thank you for taking time to talk with me today. The information you shared with me was very helpful. Is there anything you would like to ask me about? (****Pause here****).*

*Please remember that your identity is and will be completely protected and you can contact our offices at any time with questions or concerns. Thank you again for talking with me.*

**Interview conducted by:**

Full name: _____________________________________________ Initials: _________ Date: ____________ (day/month/year)
