# Supplementary material for: “The right time is just after birth”: acceptability of point-of-care birth testing in Eswatini: qualitative results from infant caregivers, health care workers, and policymakers
Source: BMC Pediatr. 2020 Jul 15;20:347. doi: 10.1186/s12887-020-02242-2 (PMC7362515; doi:10.1186/s12887-020-02242-2)
Supplement: Supplementary file 1 — Additional file 1. [file 12887_2020_2242_MOESM1_ESM.zip › Feasibility of POC BT IDI Guide_Managers_Ver 1.0 EnglishR2.docx]

**IN-DEPTH INTERVIEW GUIDE FOR LAB MANAGERS/POLICY MAKERS**

Date of Interview: ­___ ___ / ___ ___ / ___ ___ Interviewer ID:

*Day/Month/Year (e.g. 22/01/18)*

Facility Code:

Participant has signed written consent for this interview? Yes No

Participant agreed to be audio-recorded? Yes No

***Introduction:***

*“I would like to ask you about your experiences and opinions on birth testing. You can talk freely about your experience and opinions while I take notes. Please confirm if you agree to be audio-recorded. May we begin?”*

1. Tell me about your role in POC birth testing?

*[Ask the respondent to give a title of their job and day-to-day responsibilities/involvement with POC birth testing]*

1. Tell me about your experiences with POC birth testing?

*[Let the respondent describe both positive and negative experiences]*

1. Tell me about the aspects of the program (POC birth testing) which went well and those which did not go well? What should have been done to ensure that everything went well?
2. What benefits did POC birth testing bring to EID services in the country? What is now possible which was not possible before?
3. Has there been any issues of acceptance by other relevant stakeholders? These could be health facility staff, regional teams, implementing partners, national program personnel, etc.

*[Let the respondent describe the cases and reasons why there was lack of acceptance]*

1. Tell me about challenges you have experienced with the provision of POC birth testing?

[*Probe about getting consent from women, providing counselling, drawing blood, testing for results, providing results to parents, etc.]*

*[Probe on how they think of the challenges can be addressed.]*

*[Probe on staff shortages, cadres who can test, weekend issues, etc.]*

1. What has been the main barriers to providing birth testing in the country?

*[Probe for: barriers to quality, barriers to rapid/timely provision, etc.]*

*[Probe for solutions for each of the named barriers]*

1. Since the start of the pilot, have adjustments been made to the program? How have these helped or hindered the program?
2. In your observation do you think that the pilot of POC birth testing has been able to achieve its objective? Tell me why you think is has or why you don’t think it has not achieved its objective?
3. How can POC birth testing be improve in the country?
4. Do you think that Swaziland (health facilities, laboratory) is ready to provide birth testing?

Why/why not? If not, what needs to be done?

1. What are some future challenges you would see with scaling up POC EID? How can these challenges be addressed?
2. Based on your experience about POC birth testing would you recommend that the country increase the use of POC birth testing? Why would you make the recommendation or why would you not recommend that the country scale up POC birth testing?
3. What resources or other support would you need to scale up birth testing?

*[Probe for clinical, political, budgetary needs, M&E, referrals and linkages, etc.]*

1. Is there anything else you would like to share with us about your experience with birth testing?

*Thank you for taking time to talk with me today. The information you shared with me was very helpful. Is there anything you would like to ask me about? (****Pause here****).*

*Please remember that your identity is and will be completely protected and you can contact our offices at any time with questions or concerns. Thank you again for talking with me.*

**Interview conducted by:**

Full name: _____________________________________________ Initials: _________ Date: ____________ (day/month/year)
