# Supplementary material for: “The right time is just after birth”: acceptability of point-of-care birth testing in Eswatini: qualitative results from infant caregivers, health care workers, and policymakers
Source: BMC Pediatr. 2020 Jul 15;20:347. doi: 10.1186/s12887-020-02242-2 (PMC7362515; doi:10.1186/s12887-020-02242-2)
Supplement: Supplementary file 1 — Additional file 1. [file 12887_2020_2242_MOESM1_ESM.zip › Feasibility of POC BT IDI Guide_HCWs_Ver 1.0 EnglishR2.docx]

**IN-DEPTH INTERVIEW GUIDE FOR HEALTH CARE WORKERS**

Date of Interview: ­___ ___ / ___ ___ / ___ ___ Interviewer ID:

*Day/Month/Year (e.g. 22/01/18)*

Facility Code:

Participant has signed written consent for this interview? Yes No

Participant agreed to be audio-recorded? Yes No

***Introduction:***

*“I would like to ask you about your experiences and opinions on testing of infants for HIV at birth using a POC EID platform. You can talk freely about your experience and opinions while I take notes. Please remember that you do not have to answer question you do not wish to answer. May we begin?”*

1. Tell me about your experience with testing HEIs for HIV at birth.
2. *[Probe for both positive and negative experiences.]*
3. What benefits did providing birth testing bring to EID services in the health facility?
4. Do you think that providing birth testing was helpful to the mothers?
5. *[Probe on what ways it has been helpful and/not helpful]*
6. Has there been any issues of acceptance of birth testing by health facility? These could for instance colleagues, site management, etc. *[Let the respondent describe the cases and reasons why there was lack of acceptance]*
7. Do you think that the health facility was ready to provide birth testing? If not, what needed to done/ put in place before birth testing could be provided?
8. *[Probe about documentation of the provision of the services in health facility registers and log books, referrals and linkages process, training, counselling, etc.]*
9. What challenges have you experienced with providing birth testing?
10. [*Probe about getting consent from women, providing counselling, drawing blood, testing for results, providing results to parents, etc.]*
11. *[Probe on how they think of the challenges can be addressed.]*
12. *[Probe on staff shortages, cadres who can provide testing, weekend challenges, etc.]*
13. What has been the main barriers to providing birth testing in the health facility?
14. *[Probe for: barriers to quality, barriers to rapid/timely provision, etc.]*
15. *[Probe for solutions for each of the named barriers]*
16. Since the start of the pilot, were adjustments made to the program? What were they and how did they help/hinder the program?
17. Based on your experience about POC birth testing would you recommend that the country increase the use of POC birth testing? Why would you make the recommendation or why would you not recommend that the country scale up POC birth testing?
18. Is there anything else you would like to share with us about your experience with birth testing?

*Thank you for taking time to talk with me today. The information you shared with me was very helpful. Is there anything you would like to ask me about? (****Pause here****).*

*Please remember that your identity is and will be completely protected and you can contact our offices at any time with questions or concerns. Thank you again for talking with me.*

**Interview conducted by:**

Full name: _____________________________________________ Initials: _________ Date: ____________ (day/month/year)
